# Factors Influencing the Predictability and Success of Invisalign Aligners: A Systematic Review

**DOI:** 10.7759/cureus.95845

**Published:** 2025-10-31

**Authors:** Fatima Sameer AlBaqshi, Sarah Salah S Alosaimi, Amjad H Al Jumaiei, Dhuha A Alkahtani, Ali Abdullah Abu Melha Alghamdi, Raghad H Alamri, Fatema Z Ali Alali, Zainab M AlGhafli, Zahra Z Alismail, Laila Alanaz

**Affiliations:** 1 Dentistry, King Faisal General Hospital, Ministry of Health, Al Ahsa, SAU; 2 Dentistry, Taif University, Al Hawiyah, SAU; 3 Dentistry, Al-Farabi College, Riyadh, SAU; 4 Dentistry, King Khalid University, Abha, SAU; 5 Dentistry, Al Baha University, Al Baha, SAU; 6 Dentistry, Taibah University, Medina, SAU; 7 Dentistry, Vision College, Riyadh, SAU; 8 Dentistry, Ministry of Health, Riyadh, SAU; 9 Dentistry, Riyadh Elm University, Riyadh, SAU; 10 Dentistry, Private Practice, Riyadh, SAU

**Keywords:** clear aligners, invisalign, orthodontic treatment, tooth movement predictability, treatment success

## Abstract

Invisalign clear aligners have become an increasingly popular alternative to fixed orthodontic appliances due to their esthetic appeal and comfort; however, their treatment outcomes remain variable across different patient groups and movement types. This systematic review aimed to evaluate clinical, biomechanical, and patient-related factors influencing the success and predictability of Invisalign treatment. Comprehensive searches were conducted in PubMed/MEDLINE, ScienceDirect, Google Scholar and the Cochrane Library for studies published up to August 2025. Eligible studies included randomized controlled trials, cohort, case-control, and cross-sectional designs assessing factors associated with Invisalign outcomes. Six studies met the inclusion criteria. The findings revealed that treatment success is multifactorial and primarily determined by patient compliance, treatment planning, and case complexity. Males and patients without prior orthodontic experience demonstrated higher compliance rates, while inadequate wear time was a major cause of suboptimal outcomes. Tooth movement predictability varied by type, with bucco-lingual tipping being most accurate, whereas rotation, intrusion, and expansion exhibited lower precision. Technological factors also played a role, as digital monitoring tools such as the Invisalign Progress Assessment were found to overestimate tooth movement. Compared with fixed appliances, Invisalign provided shorter treatment duration and improved comfort and esthetics, but less favorable results for certain smile parameters. Meta-analysis was not feasible due to heterogeneity in study design and outcome measures. Overall, the success of Invisalign therapy depends on a complex interaction of patient behavior, treatment planning, and technological precision. Further high-quality prospective studies are needed to standardize success criteria and refine predictive tools for improved treatment outcomes.

## Introduction and background

The utilization of Invisalign® (Align Technology, Inc., Tempe, AZ, USA) clear aligners has surged in recent decades as a preferred alternative to conventional fixed orthodontic appliances, particularly for the treatment of mild to moderate malocclusions in non-growing patients. Although systematic reviews consistently conclude that Invisalign is a viable option for these cases, its efficacy diminishes when applied to more complex movements such as arch expansion, significant overbite correction, extraction space closure, and achieving precise occlusal contacts [1]. Recent clinical evidence indicates that while Invisalign can achieve satisfactory outcomes in Class I extraction cases, fixed appliances tend to yield more accurate occlusal contacts and slightly higher clinical success rates in complex tooth movements [2]. Advances in materials and biomechanical design - such as the introduction of SmartForce™ attachments and SmartTrack™ aligner materials - have enhanced the efficacy of Invisalign systems; however, the impact of these developments depends heavily on effective digital planning and biomechanical control via tools like ClinCheck® (Align Technology, Inc., Tempe, AZ, USA) [3]. One of the most unpredictable movements in Invisalign treatment is arch expansion. Clinical evidence demonstrates that expansion outcomes often fall short of predictions, with reduc­tions in efficiency from the anterior to posterior segments of the dental arch. Overcorrection and careful torque control are commonly recommended for maximizing outcomes [4]. The accuracy of specific tooth movements also varies notably by movement type. A scoping review found bucco-lingual tipping to be the most predictable, whereas rotation, intrusion, and extrusion were the least predictable movements [5]. Further, in anterior tooth rotation, the discrepancy between planned and actual rotational movement is influenced by patient age, tooth type, intended movement magnitude, and whether interproximal reduction (IPR) was performed [6]. Patient-centered factors play an equally significant role in treatment success. The patient’s past social behavior, support from caregivers or parents, and their motivation have been identified as positive predictors of overall orthodontic outcomes [7]. Finally, patient comfort and compliance remain key determinants of effective treatment. Preliminary studies using artificial neural networks (ANNs) indicate that the number of teeth with lingual attachments, lingual buttons, and upper incisor attachments are the most influential factors in predicting discomfort (pain, anxiety, quality of life) during treatment, with high predictive accuracy [8]. Despite the growing popularity of Invisalign, the literature reveals considerable variability in treatment predictability and clinical outcomes across different tooth movements, patient profiles, and treatment protocols. While several studies have explored these issues individually, the evidence remains fragmented and inconclusive. A comprehensive synthesis of the factors influencing Invisalign success is therefore essential to guide clinicians in treatment planning and patient counseling. Accordingly, this systematic review aims to critically evaluate and summarize the current evidence on the clinical, biomechanical, and patient-related factors that affect the success of Invisalign therapy, with the ultimate goal of identifying predictors of favorable outcomes and highlighting gaps for future research.

## Review

Methodology

Protocol and Registration

This systematic review was conducted in accordance with the Preferred Reporting Items for Systematic Reviews and Meta-Analyses (PRISMA) guidelines [9]. The review protocol was developed prior to the initiation of the search process to ensure methodological transparency and minimize bias.

Eligibility Criteria

Studies were considered eligible if they evaluated factors influencing the clinical success or treatment outcomes of Invisalign braces. Inclusion criteria comprised randomized controlled trials (RCTs), cohort studies, case-control studies, and cross-sectional studies published in English. Studies involving patients of any age group undergoing treatment with Invisalign clear aligners were included. Reviews, case reports, editorials, conference abstracts, and animal studies were excluded.

Information sources and search strategy

A comprehensive electronic search was carried out in major databases including PubMed/MEDLINE, ScienceDirect, Google Scholar and the Cochrane Library, covering literature published up to August 2025. Additional studies were identified by manually screening the reference lists of relevant articles. The search strategy combined keywords and Medical Subject Headings (MeSH) terms related to Invisalign, clear aligners, orthodontic treatment, treatment outcome, and success factors.

Study selection

**Figure 1:**
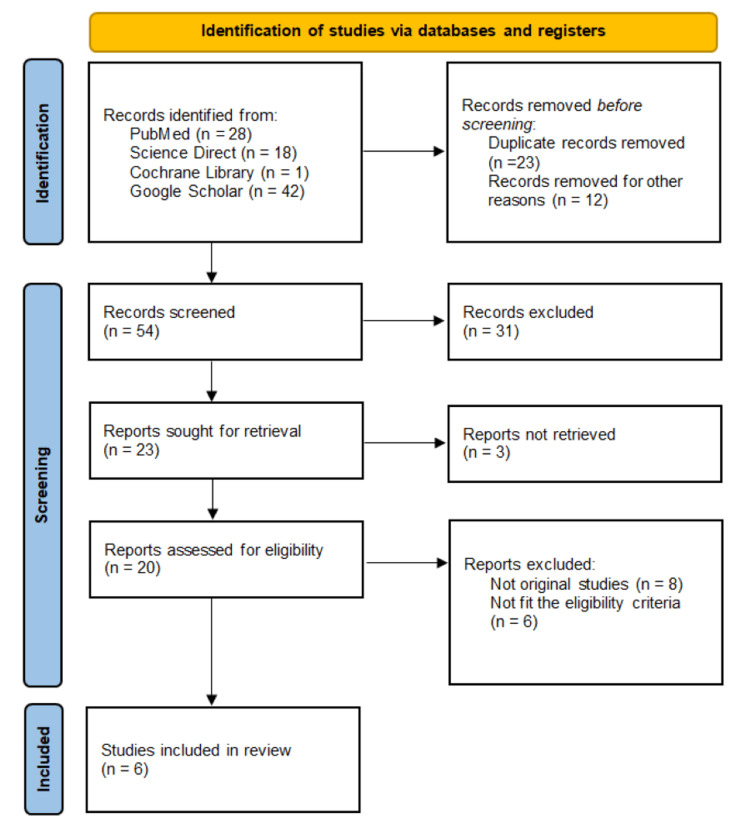
PRISMA flowchart showing the study selection process.

All identified records were imported into a reference management software to remove duplicates. Titles and abstracts were independently screened by two reviewers to determine their eligibility. Full texts of potentially relevant studies were then assessed against the predefined inclusion and exclusion criteria. Disagreements between reviewers were resolved by discussion or consultation with a third reviewer. PRISMA flowchart is shown in Figure 1.

Data extraction

Data from the included studies were extracted using a standardized form. Extracted variables included study design, sample size, patient demographics, treatment characteristics, definition and measurement of success, and reported influencing factors. Additional data on aligner compliance, complexity of malocclusion, attachment use, treatment duration, and adjunctive procedures were also collected where available.

Quality assessment

The methodological quality of the included studies was evaluated using the Newcastle-Ottawa Scale (NOS), which assesses non-randomized studies across three domains: Selection (maximum 4 stars), Comparability (maximum 2 stars), and Outcome or Exposure (maximum 3 stars) [10]. Each study was independently appraised by two reviewers, and discrepancies were resolved by discussion. Studies achieving a total score of 7-9 were considered high quality, 5-6 as moderate quality, and below 5 as low quality. The results of the quality assessment are presented in Table 1, demonstrating that most included studies were of high methodological quality, with clear inclusion criteria, representative samples, and objective outcome measurements.

**Table 1:** Quality Assessment of Included Studies Using the Newcastle–Ottawa Scale (NOS)

Study (Author, Year)	Study Design	Selection (max 4)	Comparability (max 2)	Outcome/Exposure (max 3)	Total Score (max 9)	Quality
Barashi et al., 2024 [11]	Retrospective Cohort	★★★★ (clear inclusion, representative sample, ascertainment via records, outcome not present at start)	★★ (controlled for age, malocclusion severity)	★★ (objective outcome, adequate follow-up)	8/9	High
Aref et al., 2024 [12]	Retrospective Comparative	★★★ (comparative groups clearly defined, representativeness adequate)	★★ (matched by age and malocclusion type)	★★ (objective outcome measures but unclear blinding)	7/9	High
Timm et al., 2021 [13]	Retrospective Cohort	★★★★ (large, representative sample, reliable data from app monitoring)	★★ (controlled for age, gender, and previous orthodontic history)	★★★ (objective compliance data, complete follow-up)	9/9	High
Li et al., 2023 [14]	Retrospective Laboratory	★★ (limited representativeness, small sample)	★ (no control for confounding variables)	★★★ (objective 3D measurements, reliable method)	6/9	Moderate
de-la-Rosa-Gay et al., 2025 [15]	Retrospective Cohort	★★★★ (well-defined sample, use of standardized protocol)	★★ (controlled for tooth type and arch)	★★★ (objective digital outcome measurement)	9/9	High
Christou et al., 2020 [16]	Retrospective Case-Control	★★★ (cases and controls adequately defined and comparable)	★★ (matched by age, sex, malocclusion type)	★★ (objective smile analysis but limited blinding)	7/9	High

Meta-analysis

A quantitative meta-analysis was not conducted due to the limited number of eligible studies and substantial methodological heterogeneity across them. The included studies varied considerably in design (retrospective cohort, case-control, and laboratory-based), patient populations, treatment protocols, outcome definitions, and success criteria. Additionally, most studies reported qualitative or descriptive outcomes without standardized effect measures such as mean differences or odds ratios. These discrepancies precluded data pooling and statistical synthesis. Therefore, a narrative synthesis was performed to summarize and interpret the findings in accordance with PRISMA recommendations.

Results

A total of six studies met the inclusion criteria and were incorporated into the final analysis. The included studies comprised retrospective cohort, comparative, case-control, and laboratory-based designs, with sample sizes ranging from 19 to 2,644 participants. Patient populations varied in age, sex distribution, and malocclusion type, while treatment characteristics encompassed different Invisalign protocols, adjunctive procedures, and outcome measures. Table 2 provides a summary of the key features of the included studies.

**Table 2:** Summary Characteristics of Included Studies

Study ID (Author, Year)	Study Design	Sample Size (n)	Patient Demographics (Age, Sex)	Treatment Characteristics	Definition / Measurement of Success	Key Reported Factors Influencing Success
Barashi et al., 2024 [11]	Retrospective Cohort	n=55	Age: Adults (18-60 yrs). Sex: 83.6% F, 16.4% M	Aligners: Invisalign®. Focus: Space closure cases (non-extraction or premolar extraction).	1. Need for refinement (yes/no). 2. Final space measurement (mm).	Patient-related: Male gender, Age (30-39 yrs). Treatment-related: Severe initial spacing, Longer treatment duration (≥9 months), Treatment in upper arch. Malocclusion-related: Class III Angle classification.
							Aref et al., 2024 [12]	Retrospective Comparative	Not explicitly stated (Groups: Braces & Invisalign)	Age: 12-18 yrs. Sex: Not specified.	Groups: Conventional Braces and Invisalign® clear aligners	1. Treatment duration (months). 2. Malocclusion correction success rate (%). 3. Relapse rate (%).	Treatment-related: Shorter treatment time with Invisalign (18 months vs. 24 months). Modality efficacy: Both modalities showed high success rates (88-90%) in correcting malocclusion. Stability: A non-significant trend towards higher relapse with Invisalign (12% vs. 10%).
														Timm et al., 2021 [13]	Retrospective Cohort	n=2644	Age: Adults, median 27 yrs (range 18-64). Sex: 75.0% F, 25.0% M	Aligners: Plus Dental CAT (1-1-2 protocol). Feature: Remote monitoring via mobile app.	Compliance (Primary outcome): Full: App check-in + ≥22h wear on ≥75% of aligners. Fair: ≥22h on 50-74.9% of aligners. Poor: ≥22h on <50% of aligners.	Patient-related: Higher compliance in males than females. Lower compliance in patients with a history of previous orthodontic treatment. No significant association with age or pre-treatment smile satisfaction.
Li et al., 2023 [14]	Retrospective Laboratory	n=19 (124 teeth)	Age: Mean 28.7 ± 5.35 yrs. Sex: 94.7% F, 5.3% M	Aligners: Invisalign®. Focus: Accuracy of progress assessment tool.	Accuracy of the iTero Progress Assessment tool compared to a gold standard (model superimposition on palatal rugae): 1. Tooth movement distance (mm). 2. Arch width measurements (mm).	Technology-related: The Invisalign Progress Assessment tool overestimated actual tooth movement in the horizontal plane. The tool was accurate for measuring static arch dimensions (intercanine, intermolar widths). The algorithm for model superimposition may be a source of error, as it does not use the stable palatal rugae as a reference.														
																					de-la-Rosa-Gay et al., 2025 [15]	Retrospective Cohort	n=98 (1440 teeth)	Age: Mean 42.3 ± 12.5 yrs. Sex: 70.4% F, 29.6% M	Aligners: Invisalign® (SmartTrack). Focus: Arch expansion.	Expansion Predictability: Absolute difference (mm) between planned vs. achieved expansion for canines, premolars, and molars.	Malocclusion-related: Presence of posterior crossbite (unilateral or bilateral). Tooth-related: Tooth type (less predictable in molars vs. canines), Arch (less predictable in maxilla vs. mandible). Treatment-related: Amount of predicted expansion (larger plans less predictable). Underexpansion was common (72.2% of measurements).
							Christou et al., 2020 [16]	Retrospective Case-Control	n=58 (29 Invisalign, 29 Fixed)	Age: Invisalign: 19.0 yrs; Fixed: 13.8 yrs. Sex: Invisalign: 75.9% F; Fixed: 58.6% F	Groups: 1. Invisalign®. 2. Traditional Fixed Appliances. Focus: Smile aesthetics.	Smile Outcome: Change in 15 aesthetic variables (e.g., buccal corridors, smile index, gingival display, incisor position/inclination) from pre- to post-treatment.	Treatment Modality: Fixed appliances produced significantly greater improvement in 6 smile aesthetic variables (e.g., reduced buccal corridors, improved smile index, better midline correction, ideal gingival display). Invisalign produced better outcomes for 2 variables (less proclined and less protrusive incisors). Fixed appliances were more effective overall at improving smile aesthetics for Class I non-extraction cases.														

Based on the comprehensive analysis of the included studies, a clear narrative emerges regarding the multifactorial nature of Invisalign treatment success. The evidence consistently demonstrates that clinical outcomes are influenced by a complex interplay of patient-related, treatment-related, and technology-related factors, with significant variation in predictability across different types of tooth movements. Patient compliance emerges as a fundamental determinant of success, with Timm et al. (2021) demonstrating that only 36% of patients showed full compliance with wear time recommendations (≥22 hours daily), while males and those without previous orthodontic treatment were significantly more likely to be compliant [13]. This finding is particularly crucial as inadequate wear time directly impacts the achievement of predicted tooth movement, potentially explaining why certain cases require refinements or show suboptimal outcomes.

The predictability of specific tooth movements varies substantially according to the included studies. Expansion movements demonstrate particularly variable outcomes, with de-la-Rosa-Gay et al. (2025) finding that 72.2% of measurements showed some degree of underexpansion, especially in the maxilla (absolute discrepancy of 1.24mm vs 0.61mm in mandible) and molar regions [15]. Similarly, space closure presents challenges, with Barashi et al. (2024) reporting that cases with severe spacing had a 20.9 times higher probability of requiring refinement compared to mild cases [11]. These technical challenges are compounded by limitations in monitoring technology, as Li et al. (2023) found that the Invisalign Progress Assessment tool significantly overestimated actual tooth movement in the horizontal plane, potentially leading clinicians to underestimate the need for intervention during treatment [14].

When compared to traditional fixed appliances, the evidence presents a nuanced picture of Invisalign's effectiveness. While Aref et al. (2024) reported a significantly shorter treatment time for Invisalign (18 months vs. 24 months) [12], Christou et al. (2020) found that fixed appliances produced superior improvements in most smile aesthetic parameters, including reduced buccal corridors and more ideal smile indexes [16]. This suggests that the choice between treatment modalities involves trade-offs between efficiency, aesthetic outcomes, and case complexity. Importantly, the studies indicate that success is not determined by a single factor but rather through the interaction of patient compliance, case selection appropriateness, treatment planning strategies, and the inherent limitations of the technology itself, highlighting the need for careful patient selection and management of expectations throughout treatment.

Discussion

This review demonstrates that the efficacy of Invisalign treatment is influenced by a combination of patient compliance, movement type, and biomechanical predictability. Patient compliance remains fundamental: one randomized trial found no significant difference in compliance between aligner and fixed appliance users within the first year, nor any correlation with age or gender [17]. However, a large retrospective cohort indicated that only around one-third of clear aligner users achieved full compliance (≥22 h/day), with roughly a quarter displaying poor compliance [13]. This disparity underscores that while clear aligners' wearability may seem advantageous, actual compliance varies widely and critically impacts treatment success.

Movement predictability also varied substantially. Vertical movements like intrusion and rotation often showed lower accuracy. In a prospective observational study, vestibulo-lingual tipping was highly predictable (≈93%), while rotation ranged from 86% for maxillary central incisors down to 70% for mandibular first premolars [18]. Comparatively, rotational movements - particularly of maxillary canines - demonstrated markedly low accuracy (~48%) in meta-analysis, making them one of the least predictable movements [19]. Furthermore, anterior tooth rotations were influenced by complex factors including patient age, tooth type, magnitude of planned movement, and whether interproximal reduction (IPR) was performed [6]. The influence of auxiliaries and attachment designs on aligner biomechanics has been further supported by recent network meta-analytic evidence. Khursheed Alam et al. (2024) demonstrated that auxiliaries substantially enhance the predictability of anterior root torque, rotations, and mesiodistal tooth movement, although evidence for extrusion and posterior expansion remains limited [20].

Arch expansion outcomes were similarly variable. One retrospective cohort showed maxillary expansion predictability at ~76%, with mandibular arch more accurate at ~87% [19]. Another study based on SmartTrack aligners reported high coronal-level expansion predictability (98-100%) and somewhat lower gingival-level accuracy (85-90%) - particularly in moderate expansion scenarios [21]. Systematic reviews highlight that aligner effectiveness in expansion decreases from anterior to posterior segments, and that ClinCheck planning may overestimate bodily movement, necessitating overcorrection and torque control [22].

Patient-reported outcomes strongly favor aligners for comfort and aesthetics. Improved appearance and ability to chew were among the most commonly cited benefits following Invisalign treatment [23]. Innovations in aligner materials - such as SmartTrack - have also been positively received, with users reporting significantly less pain and better overall comfort compared to earlier materials [24]. Digital advancements, too, are reshaping treatment paradigms. For instance, remote monitoring platforms like Dental Monitoring® (Paris, France) reduced in-office appointments by nearly a quarter, while maintaining equivalent treatment duration and refinement needs [22]. These findings are consistent with recent randomized evidence showing that clear aligner treatment is associated with a significantly smaller negative impact on oral health-related quality of life compared to fixed appliances, particularly in domains related to physical pain and functional limitation [25].

This systematic review has several limitations that should be acknowledged. First, the number of eligible studies was relatively small, and most were retrospective in design, which introduces inherent risks of bias and limits the ability to establish causality. Second, there was considerable heterogeneity across studies in terms of patient populations, treatment protocols, outcome definitions, and measurement methods, which precluded a quantitative meta-analysis and necessitated a narrative synthesis. Third, many included studies relied on self-reported measures of compliance or retrospective digital records, both of which are subject to reporting errors and potential overestimation of treatment progress. Fourth, the variability in aligner systems, adjunctive procedures, and clinician experience across studies may have influenced treatment outcomes but could not be standardized for comparison. Finally, the inclusion of only English-language publications raises the possibility of language bias, and the exclusion of gray literature may have resulted in missing relevant data.

## Conclusions

This systematic review highlights that the success of Invisalign treatment is influenced by a complex interaction of patient-related, treatment-related, and technology-related factors. Patient compliance with aligner wear remains the most critical determinant of achieving planned outcomes, while demographic variables such as age and sex show additional but less consistent effects. Treatment-related elements, including malocclusion complexity, severity of spacing, type of tooth movement, and treatment duration, significantly affect predictability, with certain movements such as expansion, rotation, and space closure showing greater variability. Furthermore, although digital tools have advanced monitoring and planning capabilities, limitations in accuracy underscore the need for cautious clinical interpretation. When compared with conventional fixed appliances, Invisalign demonstrates clear advantages in terms of comfort, esthetics, and reduced treatment time; however, fixed appliances often yield superior outcomes in complex cases and specific smile aesthetics. These findings suggest that careful patient selection, realistic treatment planning, and reinforcement of patient compliance are essential to optimize Invisalign outcomes. Overall, Invisalign is an effective treatment modality when used within its predictable limits. Future high-quality prospective studies are warranted to further clarify the role of emerging technologies, refine biomechanical strategies, and establish standardized definitions of treatment success. Such evidence will strengthen clinical decision-making and improve outcomes for patients seeking clear aligner therapy.

## Appendices

**Table 3:** Detailed Database Search Strategy

Database	Search String	Results (before screening)
PubMed / MEDLINE	(("Invisalign"[Title] OR "clear aligner therapy"[Title] OR "clear aligner"[Title/Abstract]) AND ("orthodontic treatment"[MeSH Terms] OR "orthodontic"[Title] OR "malocclusion"[Title/Abstract]) AND ("predictability"[Title/Abstract] OR "accuracy"[Title/Abstract] OR "treatment success"[Title/Abstract] OR "treatment outcome"[Title/Abstract] OR "tooth movement"[Title/Abstract] OR "refinement"[Title/Abstract]) NOT ("case report"[Publication Type] OR "animal"[MeSH Terms]))	28 results
ScienceDirect	(“Invisalign” OR “clear aligner” OR “clear aligner therapy”) AND (“predictability” OR “accuracy” OR “treatment outcome” OR “treatment success”) AND (“orthodontic treatment” OR “malocclusion”)	18 results
Cochrane Library	(“Invisalign” OR “clear aligner therapy”) AND (“orthodontic treatment” OR “malocclusion” OR “tooth movement” OR “treatment outcome”)	1 result
Google Scholar	Invisalign AND “clear aligner therapy” AND “predictability” AND “treatment outcome” AND orthodontic	42 results
